# l-Serine Reduces Spinal Cord Pathology in a Vervet Model of Preclinical ALS/MND

**DOI:** 10.1093/jnen/nlaa002

**Published:** 2020-01-21

**Authors:** David A Davis, Paul Alan Cox, Sandra Anne Banack, Patricia D Lecusay, Susanna P Garamszegi, Matthew J Hagan, James T Powell, James S Metcalf, Roberta M Palmour, Amy Beierschmitt, Walter G Bradley, Deborah C Mash

**Affiliations:** n1 Department of Neurology, Miller School of Medicine, University of Miami, Miami, Florida; n2 Brain Chemistry Labs, Jackson Hole, Wyoming; n3 Behavioural Science Foundation, St. Kitts and Nevis, West Indies; n4 Department of Psychiatry, McGill University, Montreal, Quebec, Canada; n5 Department of Clinical Sciences, Ross University School of Veterinary Medicine, St. Kitts and Nevis, West Indies; n6 Department of Molecular and Cellular Pharmacology, Miller School of Medicine, University of Miami, Miami, Florida; n7 Dr. Kiran C. Patel College of Allopathic Medicine, Nova Southeastern University, Davie, Florida

**Keywords:** ALS/MND, BMAA, Cyanobacteria, Guam ALS/PDC, Motor neurons, Neurofibrillary tangles, TDP-43

## Abstract

The early neuropathological features of amyotrophic lateral sclerosis/motor neuron disease (ALS/MND) are protein aggregates in motor neurons and microglial activation. Similar pathology characterizes Guamanian ALS/Parkinsonism dementia complex, which may be triggered by the cyanotoxin β-*N*-methylamino-l-alanine (BMAA). We report here the occurrence of ALS/MND-type pathological changes in vervets (*Chlorocebus sabaeus*; n *=* 8) fed oral doses of a dry powder of BMAA HCl salt (210 mg/kg/day) for 140 days. Spinal cords and brains from toxin-exposed vervets were compared to controls fed rice flour (210 mg/kg/day) and to vervets coadministered equal amounts of BMAA and l-serine (210 mg/kg/day). Immunohistochemistry and quantitative image analysis were used to examine markers of ALS/MND and glial activation. UHPLC-MS/MS was used to confirm BMAA exposures in dosed vervets. Motor neuron degeneration was demonstrated in BMAA-dosed vervets by TDP-43^+^ proteinopathy in anterior horn cells, by reactive astrogliosis, by activated microglia, and by damage to myelinated axons in the lateral corticospinal tracts. Vervets dosed with BMAA + l-serine displayed reduced neuropathological changes. This study demonstrates that chronic dietary exposure to BMAA causes ALS/MND-type pathological changes in the vervet and coadministration of l-serine reduces the amount of reactive gliosis and the number of protein inclusions in motor neurons.

## INTRODUCTION

Amyotrophic lateral sclerosis/motor neuron disease (ALS/MND) is a progressive MND characterized by rapidly progressing upper and lower motor neuron degeneration, profound muscle atrophy and paralysis, and a mean survival of 2–5 years of life after diagnosis (1). Despite significant progress in understanding the genetic basis of familial cases, 90% of these cases are sporadic in nature and poorly understood (2). While ALS/MND may be a syndrome rather than a single disease, the early neuropathological changes (3) are similar with activation of microglia, the resident macrophage population in the CNS, often presaging the eventual damage and loss of motor neurons in the spinal cord (4). Protein inclusions, particularly in anterior horn motor neurons, astrogliosis, and the loss of myelinated axons in the lateral corticospinal tracts are features of degeneration of upper and lower motor neurons (3).

The majority of the ALS/MND experimental models developed to date are based on known gene mutations (e.g. C9orf72, FUS, SOD1, and TARDBP). These animal models demonstrate neuropathological changes in motor neurons but rarely include the progressive neurodegenerative process as a whole consistent with the earliest stages of the clinical disease (5–7) or gene-environment-time interactions (8). Furthermore, these genetic mutations only represent 10% of all ALS/MND patients (9). Thus, an in vivo model, which includes the neuropathological changes that occur in presymptomatic individuals, would be useful for testing novel therapeutic strategies to prevent disease progression (10) and death of motor neurons.

The neuropathology of ALS/MND is similar to the paralytic disease suffered by the indigenous Chamorro people of Guam. Guam ALS/Parkinsonism dementia complex (PDC) includes symptoms of upper and lower motor neuron degeneration and resultant muscle atrophy, as well as protein inclusions in the spinal cord anterior horn (11). Early work suggesting that the Chamorro traditional diet was implicated in Guam ALS/PDC etiology, particularly flour made from the seeds of *Cycas micronesica* Hill (Cycadaceae) (12–14) led to the identification of the neurotoxic nonprotein amino acid β-*N*-methylamino-l-alanine (BMAA), produced by cyanobacteria in specialized roots of the cycad trees (15). BMAA was shown to accumulate in tissues of volant mammals consumed by the Chamorros (16), and to be present in tortillas and dumplings made from cycad seed flour, and thus to bioaccumulate in the brains of the Chamorros suffering from ALS/PDC (15, 16). By chronically feeding BMAA to vervets for 140 days, we demonstrated for the first time a similar neuropathology in the brain to that seen in humans with Guamanian ALS/PDC (17, 18).

BMAA is produced by all free-living cyanobacteria indicating that exposure can occur outside the Guam ecosystem (19, 20). Consumption of contaminated seafood (21–23) and living near water bodies with frequent algal blooms (24) have both been proposed as risk factors for BMAA exposure. BMAA rapidly crosses the blood-brain barrier to accumulate in the CNS and localize to neurons (25, 26). Brain uptake leads to neurotoxic injury through a variety of mechanisms, including damage to Nicotinamide adenine dinucleotide phosphate diaphorase-positive motor neurons, overstimulation of α-amino-3-hydroxy-5-methyl-4-isoxazolepropionic acid (AMPA) and N-methyl-D-aspartate (NMDA) receptors, abnormal hyperphosphorylation, mitochondrial damage, gliotoxicity, and protein misfolding (27–32). Protein misincorporation of BMAA for l-serine in SOD1 (33), albeit a relatively rare event, may lead to dissociation of the SOD1 dimer, subsequent protein misfolding, and nucleation of protein aggregates in neurons and glia (31). Thus, BMAA exposures to the brain can affect select subpopulations of motor neurons, astroglia, and microglia.

Our laboratory has previously demonstrated that chronic dietary exposure to BMAA triggers β-amyloid deposits, neurofibrillary tangles (NFTs), and glial inclusions in the cerebral cortex, including the primary motor cortex of vervets (18). In this study, we have extended our original experimental findings to examine if the same chronic BMAA exposures in the diet cause degeneration in the upper motor neuron tracts and lower motor neurons in the spinal cord. We conducted this investigation in vervets in parallel with autopsied brain and spinal cord tissues from a patient diagnosed with sporadic ALS/MND (sALS) for diagnostic neuropathology marker comparisons. We report here that chronic dietary exposure to BMAA in vervets leads to anterior horn neuron injury, TDP-43 proteinopathy, glial activation, and loss of myelinated axons in the lateral corticospinal tracts. Coadministration of l-serine reduced these neuropathological changes, protecting motor neuron injury from chronic exposure to BMAA.

## MATERIALS AND METHODS

### Toxin Dosing

Young adult male vervets (*Chlorocebus sabaeus*; age 7 years; 3.1 kg) were housed in groups inside large outdoor enclosures at the Behavioural Science Foundation (BSF) (St. Kitts, West Indies). The BSF is a fully accredited biomedical research facility with approvals from the Canadian Council on Animal Care. The BSF Institutional Animal Care and Use Committee approved the use of our experimental protocol for this study. During the dosing experiment, vervets ate a low-protein diet comprising predominantly local fruits and vegetables. For dietary exposure, l-BMAA HCl, l-BMAA HCl plus l-serine, or rice flour was placed inside a cavity of a banana and presented to vervets prior to their daily allotment of food. l-BMAA HCl salt was synthesized by Irvine Chemistry Lab (Anaheim, CA), with purity confirmed by ^1^H NMR and ^13^C NMR. Optical rotation was c = 1.15 mg/mL in 5 N HCl, +27.7°C, with a melting point of 186–190°C (Cox et al 2016 Supplementary Material). In tandem LC/MS/MS, the synthesized BMAA was consistent in mass, product ions, and product ion ratios with an authenticated standard (B-107, Sigma-Aldrich, St. Louis, MO). Vervets were randomly assigned to one of three 8-member cohorts for 140 days of dosing with l-BMAA HCl at 210 mg/kg/day, l-BMAA HCl plus l-serine both at 210 mg/kg/day, and a control cohort dosed with 210 mg/kg/day of rice flour. The 140-day BMAA dosing regimen of the adult vervet was calculated to be equivalent to the 20-year lifetime exposure of an adult Chamorro male consuming a 30-g serving of cycad powder per day in tortillas and 8 flying foxes (*Pteropus mariannus*) per month (17). Group enclosures for each vervet cohort were spaced appropriately apart to reduce the chance for sharing bananas to cause cross contamination. The 210 mg/kg of powdered l-BMAA HCl delivered an effective daily dose of 161 mg/kg l-BMAA HCl (17, 18). The doses were prepared at Brain Chemistry Labs (Jackson, Wyoming) using a Mettler Toledo balance with a Quantos automated powder-dispensing module at a tolerance of ±0.1% of target dose. To ensure oral delivery of test substances, veterinarian, BSF and Brain Chemistry Labs staff monitored daily dosing. Cerebral spinal fluid, hair, and plasma were sampled every 4 weeks from each vervet under ketamine anesthesia, and body weight was recorded. All vervets were observed daily for mortality, morbidity, and clinical signs of adverse health effects and qualitative food consumption.

### Necropsy and Histopathology

After the 140-day, chronic dosing regimens, vervets were euthanized under ketamine anesthesia followed by complete dissection of brain and spinal cord tissues. All the external surfaces of specimens were examined, photographed, and fixed in 10% neutral buffered formalin for histopathological studies. Brain tissues were placed into phosphate buffered saline (PBS), pH 7.4, and shipped to NeuroScience Associates (Knoxville, TN) for processing, embedding, and sectioning into 40 μm sections on 76 × 51 mm glass slides using the MultiBrain Service. Spinal cords were processed using xylenes and alcohols and transverse segments were embedded in paraffin wax in preparation for Leica microtome sectioning into 7-μm sections on 75 × 25 mm glass slides. Slide mounts of 3 cervical and 3 lumbar spinal cord segments were deparaffinized in 3 changes of xylene for 10 minutes each, followed by 2 changes of absolute ethanol for 5 minutes each, then 95% ethanol for 5 minutes. Routine histological stains included hematoxylin and eosin (H&E), periodic acid-Schiff, luxol fast blue, thionine-Nissl, and thioflavin-S. To probe for specific protein antigens after hydration, slide tissue mounts were incubated in 3% H_2_O_2_ in methanol for 10 minutes followed by rinsing in distilled water for 3 changes of 5 minutes. Slides were then incubated in antigen retrieval buffer (98% formic acid for 45 seconds or citrate buffer for 30 minutes, where applicable), followed by a wash in 3 changes of distilled H_2_O on a Thermolyne Roto Mix shaker and incubation in PBS for 5 minutes. To block nonspecific antibody binding, 10% normal donkey serum (NDS) in PBS was applied to slides in a humidity chamber and incubated at room temperature for 30 minutes. Brain and spinal cord slide mounts were probed with antibodies against β-amyloid (A_**β**_: 1:800, Covance, Princeton, NJ), cluster of differentiation 68 (CD68: 1:500, DAKO/Agilent, CA), fused in sarcoma (FUS: 1:2000, Novus Biological, Centennial, CO), ionized calcium binding adaptor molecule 1 (IbA1: 1:300, FUJIFILM Wako Chemicals, Richmond, VA), glial fibrillary acidic protein (GFAP: 1:1000, Sigma-Aldrich), phospho-tau (Ser202, Thr205) monoclonal antibody (AT8: 1:3000, Thermo Fischer Scientific, Waltham, MA), phosphorylated TAR DNA-binding protein 43 (TDP-43: 1:1000, Cosmo-Bio, Carlsbad, CA), and ubiquitin (Ubiq: 1:300, Millipore, Waltham, MA). Slides were incubated with antibodies at 4°C overnight. Slides were rinsed with PBS for 3 changes of 10 minutes, followed by additional application of 2% NDS for 10 minutes prior to incubation with secondary antibodies. A donkey anti-mouse biotin (1:200; Jackson Immunoresearch, West Grove, PA) conjugated secondary antibody goat anti-mouse/or anti-rabbit was incubated on tissue sections for 2 hours at room temperature, rinsed with a PBS wash for 10 minutes, and followed by application of ExtrAvidin peroxidase (1:5000, Sigma-Aldrich) in PBS for 1 hour. ExtrAvidin peroxidase was detected using diaminobenzidine (DAB) solution (100 mL DAB = 98 mL PBS + 2 mL 25 mg/mL DAB + 16.6 μL 3% H_2_O_2_) for 10 minutes. Slides were washed in 2 changes of PBS, rinsed with distilled water, and counterstained with Gill No. 1 hematoxylin for 20 seconds and rinsed under running tap water for 5 minutes. Phospho-Tau AT8 immunohistochemistry was performed on cortical brain sections by NeuroScience Associates. Sevier Münger silver staining was performed at AML Laboratories (Jacksonville, Florida) and counterstained with Gill No. 1 hematoxylin. Postmortem spinal cord and brain tissues from a 58-year-old Caucasian male with a neuropathologically confirmed diagnosis of sALS were used as positive controls with each immunostaining protocol.

### Digital Pathology and Microscopy

Histology slides were scanned at 40× resolution using a TissueScope LE (Huron Digital Pathology, Waterloo, Ontario, Canada). Digital scanning allowed for complete mapping of entire spinal cord sections and clear visualization of margins and anatomical landmarks at an optimal resolution of (0.2 μm/pixel [Px] at 40×) for image analysis. High quality tiff image file (1721 × 985 Px or 3259 × 1174 Px) fields were exported from TissueScope LE and imported in NIH ImageJ 64 VER1.44o (NIH, Bethesda, MD) for analysis. To determine the total area, number, and size of glial cells and neurons, 12 tiff images were analyzed per vervet (6 fields per cervical and 6 fields per lumbar spinal cord segments) (Supplementary Fig. S1). Spinal cord tissue sections were blinded and scored manually and compared to automated counting. For automated analysis, an unbiased threshold was applied to regions of interest to determine the morphological findings across treatment groups (Supplementary Fig. S1). Slides stained with thioflavine-S and 4′,6-diamidino-2-phenylindole (DAPI) were visualized at 20× magnification using a Zeiss Apotome fluorescent microscope (Thornwood, NY).

### Detection and Quantification of BMAA Toxin

Spinal cord tissues were analyzed to measure the concentration of BMAA using triple quadrupole tandem mass spectrometry (UHPLC-MS/MS) with a precolumn 6-aminoquinolyl-*N*-hydroxysuccinimidyl carbamate (AQC) derivatization employing a method validated according to peer AOAC International guidelines and previously reported (17, 34) (Supplementary Fig. S2).Spinal cord samples (50 mg) were extracted with Trichloroacetic Acid (TCA) (20% w/v) for free BMAA followed by HCl (6.0 M) hydrolysis of the pellet at 110°C for 16–18 hours to release protein-bound BMAA. The presence of BMAA in the supernatant after TCA extraction was checked by further HCl hydrolyzation of the supernatant. Extracted samples were filtered using a centrifuge filter (0.2 μm, Millipore UltrafreeMC) at 14 000*g* for 5 minutes and analyzed on a Thermo Scientific TSQ Quantiva triple quadrupole mass spectrometer attached to a Thermo Vanquish Ultra-High Pressure Liquid Chromatography Autosampler equipped with a Vanquish pump and column compartment. Separation of 4 BMAA structural isomers (*N*-[2-aminoethyl] glycine, 2,3-diaminobutanoic acid, 2,4-diaminobutyric acid, and β-amino-*N*-methyl-alanine) was achieved with gradient elution using 20 mM ammonium acetate, pH 5.0 (A) and 100% methanol (B) as follows: 0.5 mL/minutes, 0 minutes 10% B, 1.0 minutes 10% B, 4.8 minutes 40% B (curve 5), 5.0 minutes 90% B (curve 5), 6.8 minutes 90% B, 6.81 minutes 10% B (curve 5), and 8 minutes 10% B. Separation was performed using a Thermo Hypersil Gold C-18 column (PN 25002-102130) 100 × 2.1 mm, particle size 1.9 μm heated to 65°C. Samples were analyzed in positive ion, single reaction monitoring mode using heated electrospray ionization using previously published ion transitions (18). Mass spectrometer ion source properties were as follows: 3500 V-positive ion, 45 Arb Sheath gas, 10 Arb Aux gas, Sweep gas 0.1 Arb, vaporizer temperature 400°C, and ion transfer tube temperature 350°C. Validation curves and parameters were performed as in Glover et al (34) passing all criteria exceeding minimum requirements for a single-laboratory validation. Limit of Detection (0.009 ng/mL) and Lower Limit of Quantification (0.037 ng/mL) were calculated according to U.S. Food and Drug Administration recommended regulatory guidelines (35). All samples were run and normalized with an internal BMAA standard (β-*N*-methyl-*d*_3_-amino-dl-alanine-^15^N_2_) at a concentration of 1.5 ng/mL. System blanks (AQC derivatized blanks, internal standards, and deionized water) were injected between sample injections.

### Statistical Analyses

Prism Version 7 software (Graph Pad, La Jolla, CA) was used to perform statistical analyses. Single comparisons tests were analyzed using Student *t* test and Mann-Whitney *U* test. Multiple comparisons were analyzed using one-way analysis of variance (ANOVA) with Newman-Keuls Multiple Comparison test, the Friedman with Dunn’s multiple comparison tests, or the Wilcoxon match pairs sign test. For correlation analyses, Pearson’s correlation coefficient and Spearman correlation tests were used to determine significance. D’Agostino and Pearson test was used to determine normality of each data set. All data were expressed as the median ± standard error; significance level of α = 0.05. Due to the limited size of our sample cohort, Cohen’s f was used to determine effect size. A table of descriptive statistics for each statistical comparison can be found in the Supplementary Table S1.

## RESULTS

### Toxin Accumulation

Spinal cord tissues collected at necropsy from BMAA and BMAA + l-serine cohorts were both positive for the cyanobacterial toxin (Table 1 and Supplementary Fig. S2). As expected, the rice flour control group had no detectable levels of BMAA (Table 1). The median total BMAA concentration in spinal cord tissues was 61.4 μg/g ± 13.7 SE and ranged from 15.8 to 199.9 μg/g in the BMAA and BMAA + l-serine dosing cohorts. The median and range of concentration of detectable BMAA in vervets were similar to those measured in cortical brain tissues from individuals with Guam ALS/PDC (36) and spinal cords from North Americans with ALS/MND (37). The concentrations of free and protein-bound BMAA were positively correlated (r = 0.919, p *<* 0.0001). In addition to these two protein fractions, considerable BMAA was found in the hydrolyzed supernatant indicating small soluble peptides present in spinal cord tissues (Table 1). Coadministration of l-serine did not significantly reduce the median concentration of BMAA, in any fraction of the vervet spinal cord (Table 1 and Supplementary Table S1).

**TABLE 1.** Detection of Free and Protein-Bound BMAA in Vervet Spinal Cord

Exposure Cohort	Free (μg/g)	Hydrolyzed Protein Pellet (μg/g)	Hydrolyzed Supernatant (μg/g)
Rice flour	ND	ND	ND
BMAA	8.9 ± 3.9^**NS**^	0.5 ± 0.3^**NS**^	34.0 ± 20.5 ^**NS**^
BMAA + l-serine	9.4 ± 2.2	0.6 ± 0.1	56.2 ± 13.6

Median ± SEM of BMAA per gram of spinal cord tissues; ND, not detected; NS, no statistically significant differences were found between groups using a nonparametric Mann-Whitney *U* test.

### Anterior Horn

**FIGURE 1.**
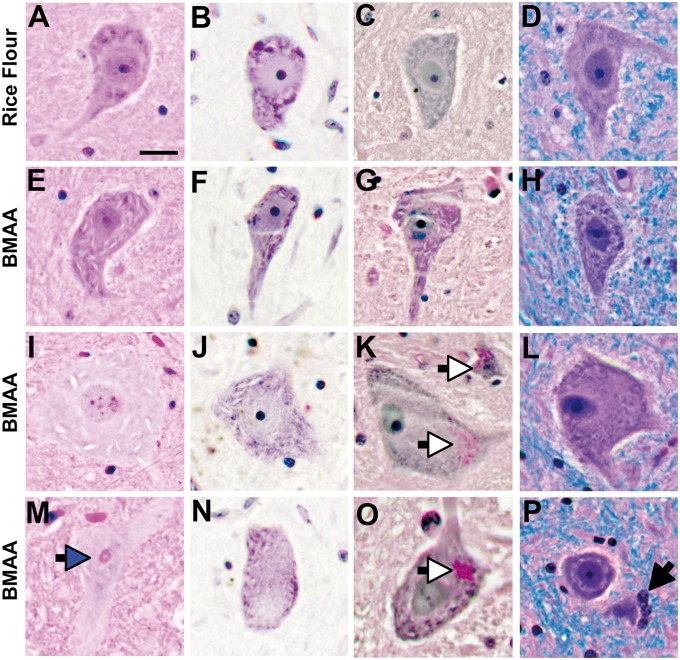
Motor neuron injury. **(A–D)** Vervets dosed with rice flour (210 mg/kg/day) for 140 days displayed anterior horn motor neurons with normal cytoarchitecture. Vervets dosed with the cyanotoxin BMAA (210 mg/kg/day) for 140 days, at an equivalent quantity to that of a 20-year exposure received by a Chamorro of Guam (17), exhibited shrunken, eosinophilic motor neurons **(E)** with thinning Nissl substances **(F)**, metabolic stress **(G)**, and abundant skeinlike cytoplasmic vacuoles **(E-P)**. **(I–L)** A small proportion of anterior horn motor neurons were observed to have enlarged cell somas with pale cytoplasm, expanded cytoplasmic vacuoles and thinning of Nissl substances. In addition, Bunina bodies (blue arrow) **(M)**, chromatolysis **(K**, **L**, **N)** glycogen cytoplasmic deposits (white arrows) **(K**, **O)** and cell death with neuronophagia (black arrow) **(P)** were observed. Scale bar: 40 μm.

**FIGURE 2.**
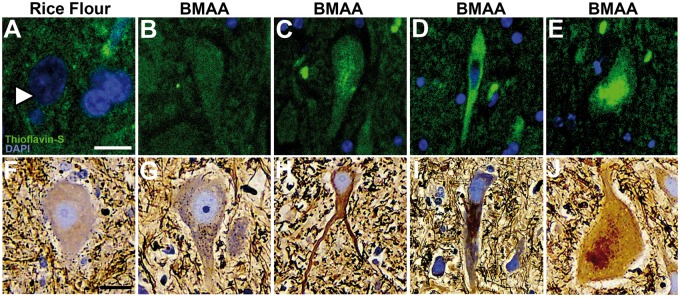
Dystrophic and argyrophilic motor neurons. Illustrative images of thioflavin-S/DAPI and Sevier Münger/hematoxylin-stained anterior horn motor neurons from vervets dosed with rice flour (210 mg/kg/day) or BMAA (210 mg/kg/day) for 140 days. Neurons from rice flour-dosed cohorts were negative for thioflavin-S fluorescence and Sevier Münger staining **(A**, **F)**. In BMAA-dosed cohorts thioflavin-S and Sevier Münger staining displayed mild to moderate insoluble and argyrophilic intraneuronal inclusions in motor neurons ranged from diffused **(B**, **G)**, filamentous **(C**, **H)**, to dense **(D**, **I)** and granular types **(E**, **J)**. Scale bar: 25 μm **(A)** and 40 μm **(B–J).**

Microscopic examination of lower motor neurons of the cervical spinal cord anterior horns of BMAA-dosed vervets showed 4.3-fold greater frequency of eosinophilic neurons (p *=* 0.024) that were reduced 40% in size (p ≤ 0.0001) and contained abundant skeinlike cytoplasmic vacuoles (p *=* 0.0002) that were absent in controls dosed with rice flour (Fig. 1A–H and Supplementary Fig. S3). Anterior horn neuronal numbers were decreased by 23% (p *=* 0.0016) and a portion was observed to have thinning of Nissl substances and chromatolysis (Fig. 1I–L, N and Supplementary Fig. S3 ). Bunina bodies, a pathological hallmark of ALS/MND (38) and Guam ALS/PDC (11), were also observed in spinal cord motor neurons of vervets dosed with BMAA (Fig. 1M). The accumulation of glycogen (Fig. 1K, O), a sign of metabolic dysfunction (39), along with cell death and neuronophagia was also present in anterior horn neurons (Fig. 1P). Other qualitative markers of ALS/MND-type cellular injury included the intracellular accumulation of thioflavin-S^+^ inclusions (40) and dystrophic argyrophilic neurons similar to NFTs (41) were abundant in the BMAA-dosed cohort, but not observed in rice flour-fed primates (Fig. 2). Extracellular amyloid beta^+^ plaques were not observed in the spinal cords of any cohort.

**FIGURE 3.**
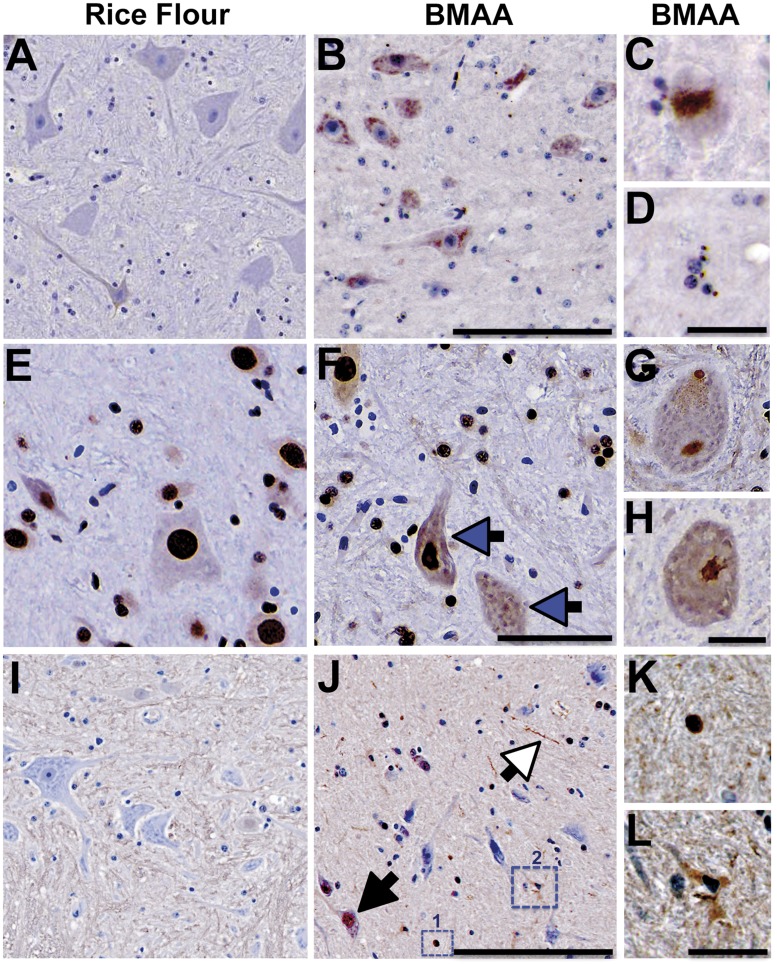
Proteinopathies. **(A)** Representative microscopic images of spinal cord anterior horn motor neurons from control vervets immunoprobed with anti-TDP-43. **(B)** Classic granular phosphorylated TDP-43^+^ intracytoplasmic inclusions were observed in anterior horn motor neurons of vervets dosed with the BMAA neurotoxin (210 mg/kg/day) for 140 days. **(C**, **D)** Dense, rounded, and spicule-like TDP-43^+^ cytoplasmic inclusion bodies were also observed. **(E)** Motor neurons of rice flour controls displaying predominant nuclear staining of FUS protein, whereas, **(F)** a small fraction of BMAA-dosed vervets displayed increased cytoplasmic translocation of FUS^+^ nuclear protein (blue arrow). **(G**, **H)** Representative high-powered images of anterior horn motor neurons with pathological cytoplasmic FUS^+^ inclusions. **(I)** Anterior horn motor neurons from rice flour control vervets probed with anti-UBIQ. **(J–L)** ALS/MND-type UBIQ^+^ inclusions were present in neuronal cell bodies and nuclei (black arrow), neurites (white arrow), and glia were observed in BMAA-dosed vervets. **(K)** High magnification of a dense UBIQ^+^ deposit shown in small dotted square #1 in **(J)** and **(L)** high magnification of a UBIQ^+^ microglia from large dotted square #2 in **(J)**. Scale bars: 250 μm **(A**, **B**, **E**, **F)**, 50 μm **(C**, **D**, **G**, **H**, **K**, **L)**, and 300 μm **(I**, **J).**

Protein inclusions in anterior horn cells typically associated with the diagnosis of ALS/MND were also found in the BMAA-dosed vervet cohorts. Neurons from 12 of 16 (75%) of vervets fed BMAA were positive for TDP-43 dense, granular, and round cytoplasmic inclusion bodies (Fig. 3A–D and Table 2) (42). The mean density and distribution of TDP-43^+^ inclusions in toxin-dosed vervets were 5-fold greater (p *=* 0.024) than those observed in controls, which is similar to that observed in Guam ALS/PDC (43) (Table 2). Rare cytoplasmic mislocalization of FUS^+^ protein, a marker used in postmortem diagnosis of TDP-43-negative ALS/MND patients (44), was observed in 2 of the 16 (12.5%) of BMAA-dosed vervets (Fig. 3E–H). Anterior horn neurons and glia also displayed UBIQ^+^ cytoplasmic inclusions and neurites (Fig. 3I–L). The presence of both TDP-43^+^ and UBIQ^+^ protein inclusions along with Bunina bodies in anterior horn neurons suggests that chronic BMAA dosing causes motor neuron injury in vervets characteristic of ALS/MND and Guam ALS/PDC (45).

**TABLE 2.** Quantitative Analysis of pTDP-43^+^ Anterior Horn Motor Neurons

Exposure Cohort	Cervical Segment	Lumbar Segment	All Spinal Cord Segments
Rice flour	0.0 ± 0.8	0.0 ± 0.8	0.0 ± 0.7
BMAA	3.0 ± 1.9	3.0 ± 3.1	4.5 ± 1.7*
BMAA + l-serine	3.0 ± 2.8	4.5 ± 3.6	3.8 ± 1.1^**NS**^

Median ± SEM of the number of TDP-43^+^ motor neurons per region of interest. n = 8 (rice flour vs BMAA); NS, no statistically significant differences were found using the Mann-Whitney *U* test.

* p *=* 0.02.

**FIGURE 4.**
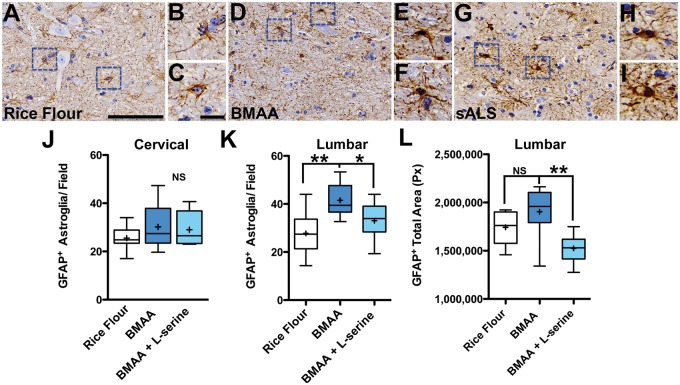
Reactive astrogliosis. **(A)** GFAP^+^ astroglial cell bodies and processes in rice flour-dosed controls (210 mg/kg/day) were compact and proximate to intact anterior horn motor neurons. **(B, C)** High-powered digital pathology scans of astroglia labeled in blue dotted squares. **(D–F)** Severe reactive GFAP^+^ astroglia were observed adjacent to shrunken and vacuolated anterior horn motor neurons in vervets dosed with the BMAA toxin (210 mg/kg/day) for 140 days. **(G–I)** Comparative digital pathology scans of GFAP^+^ astroglia from a patient with sporadic ALS (sALS). **(J)** BMAA dosing had mild effects on the cervical spinal cord (^**NS**^, p = 0.427; n = 8). **(K)** However, BMAA dosing significantly increased the median numbers of GFAP^+^ astroglia in the anterior horn of the lumbar spinal cord by 44% (**p = 0.008; n = 8). Coadministration of l-serine reduced the numbers of the GFAP^+^ astroglia by 21%. **(L)** Automated image analysis of the anterior horn show that BMAA effects were reduced 20% by coadministration of l-serine (**p = 0.0078; n = 8). Scale bars: 100 μm **(A**, **D**, **G)** and 25 μm **(B**, **C**, **E**, **F**, **H**, **I).**

Astroglia are nonneuronal cells that support neuronal plasticity, modulate synaptic transmission and have been previously shown to be susceptible to BMAA toxicity (25, 46, 47). In our study, vervets receiving fruit supplemented with rice flour displayed only astroglia with normal cellular architecture that were distributed adjacent to healthy motor neurons in the spinal cord (Fig. 4A–C). Chronic dietary dosing with BMAA increased the density of GFAP^+^ activated astroglia 1.4-fold (p *=* 0.008) in the vicinity of atrophic and vacuolated motor neurons in the anterior horns of the lumbar spinal cord (Fig. 4D–F, K). GFAP^+^ astroglia in BMAA-dosed vervets displayed morphological changes similar to those found in a sALS patient (Fig. 4G–I). Reactive astrogliosis was also observed in the primary motor cortex and midbrain with decreasing density progressing down the neuroaxis of toxin-dosed vervets (Supplementary Fig. S4A–D). Coadministration of l-serine reduced the total number (p *=* 0.008) and total area (p *=* 0.007) of GFAP^+^ astroglia in the anterior horn of the lumbar spinal cord by 21% and 20%, respectively (Fig. 4K, L).

### Lateral Corticospinal Tract

**FIGURE 5.**
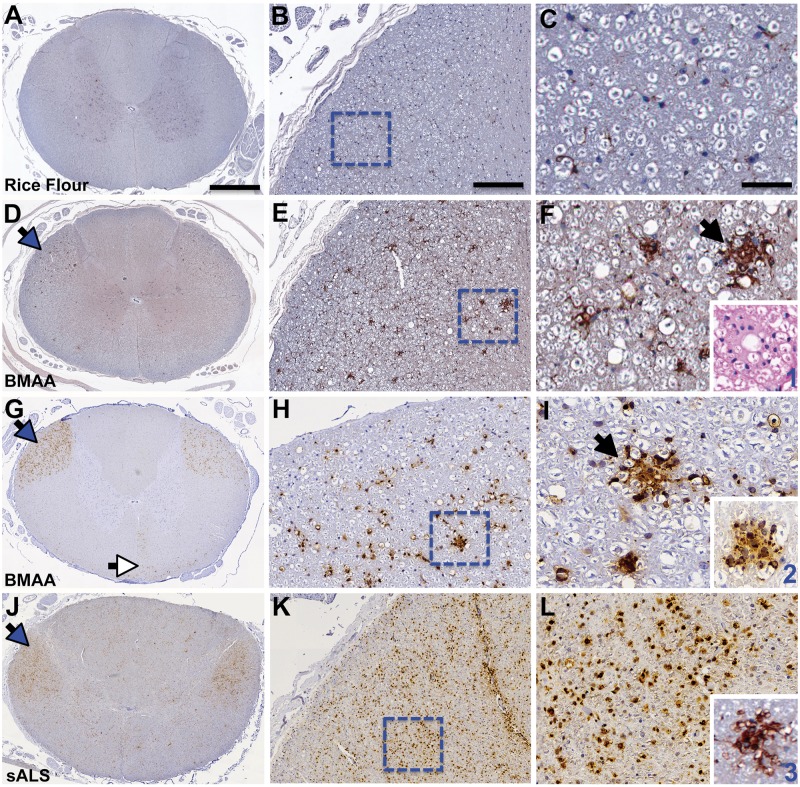
Microglial activation. **(A-C)** Vervets dosed with rice flour (210 mg/kg/day) for 140 days had a normal distribution of quiescent microglia in anterior and posterior horns and both ascending and descending white matter tracts of the cervical spinal cord. **(D)** Vervets dosed with the cyanobacterial toxin BMAA (210 mg/kg/day) displayed an increased density of activated IbA1^+^ microglia in ascending motor neuron pathways (blue arrow). **(E)** Large focal and bilateral nodules of IbA1^+^ microglia were observed in the lateral corticospinal tracts (dotted squares). **(F)** Microglial nodules (black arrow) varied in sized and distribution adjacent to a vacuolated neuropil. (Insert 1) A microglial nodule visualized with H&E staining. **(G)** Corticospinal tracts, lateral (blue arrow) and anterior (white arrow), were positive for CD68, a marker for proinflammatory microglial activation. **(H)** Large focal nodules of CD68^+^ microglia were observed (dotted square). **(I)** Predominate focal (black arrow) and foamy phagocytic (Insert 2) microglial nodules were present in BMAA toxin-dosed cohorts. **(J–L)** A patient with sporadic ALS (sALS) displaying predominately diffused CD68^+^ microglial immunostaining evident of advanced disease stage. (Insert 3) Displays an Iba1^+^ microglial nodule from a sALS patient. Scale bars: 1200 μm (left panel **A–G**), 1500 μm (left panel **J**), 500 μm (middle panel **B–K**), and 250 μm (right panel **C–L**).

**FIGURE 6.**
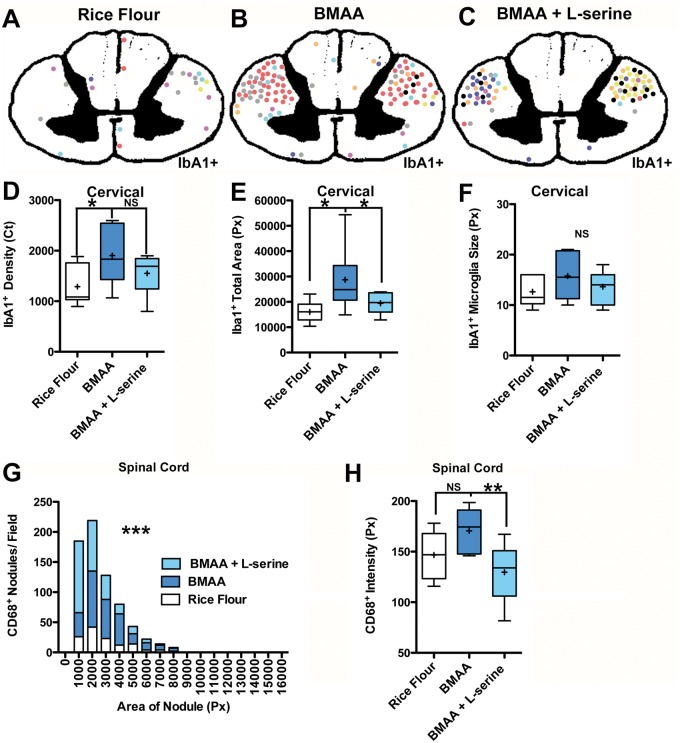
l-Serine neuroprotection. **(A–C)** Topographical maps illustrating manual regional analysis of microglial activation in the cervical spinal cord for rice flour, BMAA, or BMAA + l-serine-dosed vervets. Each vervet is identified by a unique colored dot representing a large area of Iba1^+^ microglial activation or nodule. BMAA-dosed vervet cohorts had increased bilateral microglial activation in the lateral corticospinal tracts. Adjacent anatomical regions analyzed were unaffected. To determine the therapeutic effects of l-serine, automated image analyses were performed on lateral corticospinal tracts of each dosing cohort. **(D)** Chronic dietary dosing with BMAA (210 mg/kg/day) for 140 days increased the median density (pixel counts) by 1.7-fold (*p = 0.048; n = 8) and **(E)** the total area of the lateral corticospinal tracts covered by IbA1^+^ microglia by 1.6-fold (*p = 0.011; n = 8). **(F)** BMAA dosing did not have a significant effect on the size of Iba1^+^ microglia in the lateral corticospinal tracts (NS, p = 0.260; n = 8). **(G)** The distribution of CD68^+^ microglial nodule number and size (***p < 0.0001; n = 8) was increased in the cervical and lumbar (spinal cord) segments of toxin-exposed primates. **(H)** Coadministration of l-serine also reduced the effects of BMAA on CD68^+^ microglial nodule numbers, size, and expression intensity (**p = 0.01; n = 8).

Microglia are resident macrophage/immunocells of the central nervous system that play an important role in synaptic regulation and modulation of neuron networks, as well as inflammation and scavenging. Microglial activation is one of the earliest pathological changes in ALS/MND (48). Chronic dietary dosing with BMAA increased the density 1.7-fold (p *=* 0.048) and total area 1.6-fold (p = 0.011) of IbA1^+^ microglia in the cervical spinal cord of vervets compared to controls fed rice flour (Figs. 5A–F and 6A–F). BMAA effects on IbA1^+^ microglia in the spinal cord were region-specific and targeted predominantly to descending motor pathways. Ascending white matter tracts such as the dorsal column, the spinocerebellar, and the anterolateral system were unaffected (Figs. 5D and 6B, C and Supplementary Fig. S1). IbA1^+^ microglia were observed forming large nodule-type lesions (49) and phagocytes in the lateral corticospinal tracts of BMAA-dosed vervets, but not in rice flour controls (Figs. 5A–F and 6A–F). The density and the total area covered by IbA1^+^ microglia were 1.5-fold (p = 0.0039) greater in the cervical compared to the lumbar segments of the spinal cord from BMAA-dosed vervets (Table 3). Iba1^+^ microglia nodules were also observed in the pyramids of the medulla, the cerebral peduncles, and cortical areas of BMAA-dosed vervets (Supplementary Fig. S4E–H).

**TABLE 3.** Quantitative Analysis of IbA1^+^ Microglia in the Lateral Corticospinal Tracts

	Total Area (Px)	IbA1^+^ Density (Ct)	Size (Px)
Cervical Segment			
Rice flour	16 067.5 ± 1442.3	1084.3 ± 138.8	11.9 ± 1.1
BMAA	24 839.4 ± 4357.2*	1831.8 ± 206.9*	15.4 ± 1.7^**NS**^
BMAA + l-serine	19 719.4 ± 1464.9*	1691.1± 137.3^**NS**^	14.1 ± 1.1
Lumbar segment			
Rice flour	15 285.4 ± 1336.9	1398.3 ± 257.3	14.6 ± 7.5
BMAA	17 092.1 ± 2194.3^**NS**^	1213.7 ± 151.5^**NS**^	13.9 ± 1.9^**NS**^
BMAA + l-serine	12 379.3 ± 1388.1	963.9 ± 152.2	11.8 ± 4.8

Median ± SEM of IbA1^+^ microglia automated measurements. n = 8 vervets per group; ANOVA test (cervical segments); NS, no statistical significance using Kruskal-Wallis test (lumbar segments); Ct, counts; Px, pixels.

* p ≤ 0.05.

**FIGURE 7.**
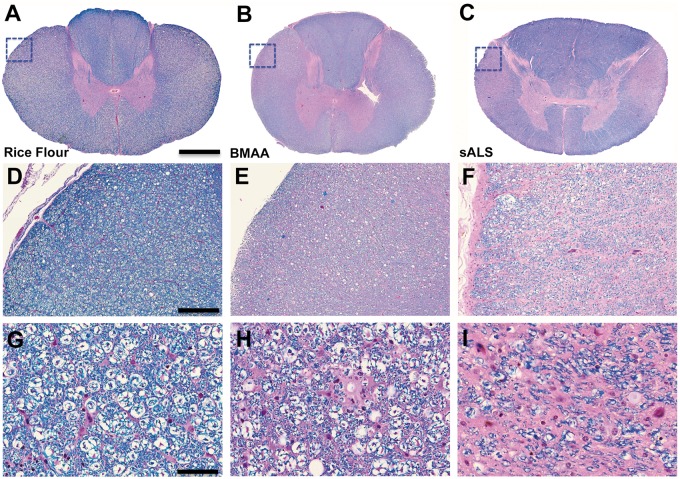
Myelinated axon fibers. **(A**, **D**, **G)** Digitized pathology scans of a Luxol fast blue (LFB) counterstained with hematoxylin and eosin (H&E) spinal cord segment shown in low (3.3×), medium (11×), and high power (40×) from a vervet dosed with rice flour for 140 days. **(B**, **E**, **H)** Vervets cohorts dosed with the cyanotoxin, BMAA (210 mg/kg/day), for 140 days displayed pallor of staining of myelinated axon fibers in the lateral corticospinal tracts (dotted square). Loss of myelinated axon fiber staining was observed in 7 of 16 (44%) (NS, p = 0.0956; n = 8) of vervets exposed to the toxin. **(C**, **F**, **I)** Representative images of LFB/H&E-stained spinal cord segment from an individual with sALS for comparison. Scale bar: 1300 μm **(A**, **B)**, 1900 μm **(C)**, and 400 μm **(D–F).**

CD68, a marker of proinflammatory microglial activation, was bilaterally expressed in the lateral corticospinal tracts in both cervical and lumbar segments of the spinal cord of BMAA-dosed vervets (Fig. 5G–I). CD68^+^ microglial density and distribution observed in BMAA-dosed vervets were similar to those seen in a representative autopsy specimen from an individual with neuropathologically confirmed sALS (Fig. 5J–L). CD68^+^ nodules had increased number and size (p ≤ 0.0001) compared to those of control vervets (Figs. 5G–I and 6G). In addition to proinflammatory microglial activation, we observed pallor of myelin staining in the pyramidal tracts suggesting mild loss of myelinated axon fibers in the lateral corticospinal tracts in 7 of 16 (44%) of vervets fed BMAA (p = 0.0956) (Fig. 7 and Supplementary Table S1). The mean Iba1^+^ microglia distribution and CD68^+^ microglial expression in the lateral corticospinal tract stimulated by BMAA dosing was attenuated 32% and 24%, respectively by coadministration of l-serine in the diet (Fig. 6 and Table 3).

### Corticospinal Pathology

**FIGURE 8.**
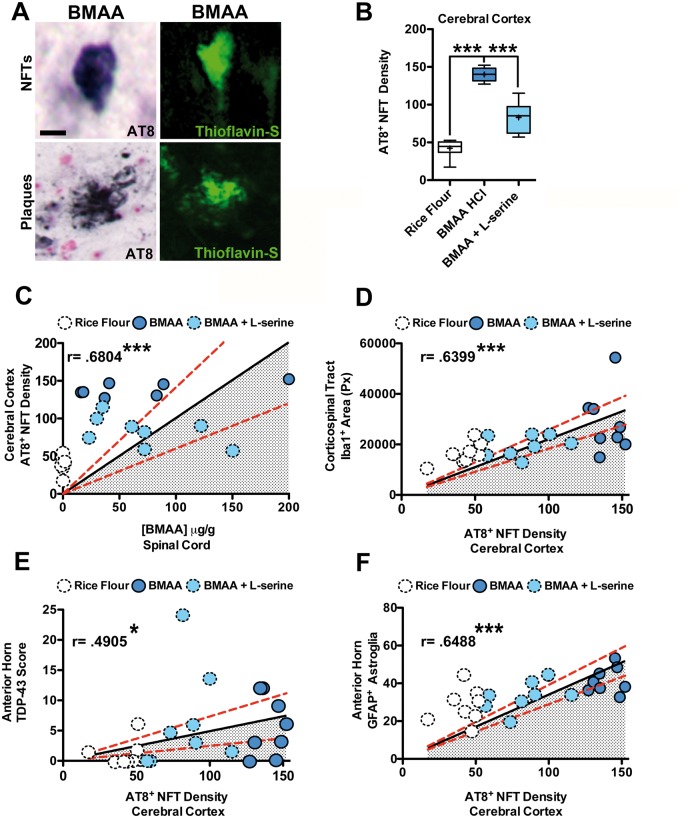
Corticospinal neuropathology. **(A)** Representative digital pathology scans of an NFT and a neuritic plaque observed in the cerebral cortex of vervets fed with BMAA (210 mg/kg/day) for 140 days. **(B)** The median cortical tau AT8^+^ NFT density was calculated by averaging automated density counts across the following seven brain regions: cingulate, entorhinal, frontal, insular, motor, occipital, and temporal cortices. BMAA dosing increased the median density of AT8^+^ NFTs 3.1-fold (***p *<* 0.0001; n = 8). The production of BMAA-induced tauopathy was partially blocked by coadministration of l-serine. **(C)** Cortical AT8^+^ NFT deposition in BMAA-dosed vervets was positively correlated with detectable levels of total BMAA in spinal cord tissues (r = 0.6804; ***p *=* 0.0004; n = 23); **(D)** the total area of IbA1^+^ microglia in the lateral corticospinal tract (r = 0.6399; ***p *=* 0.0003; n = 24); **(E)** the number of TDP-43^+^ cytoplasmic inclusions in anterior horn motor neurons (r = 0.4905; *p *=* 0.01; n = 24); and **(F)** the number of GFAP^+^ astroglia adjacent to motor neurons (r = 0.6488; ***p *=* 0.0004; n = 24). Coadministration of l-serine with BMAA reduced the observed pathology in the cerebral cortex and spinal cord of BMAA-dosed vervets. The solid black lines on scatter plots represent the best-fit line at 95% confidence interval. The red dotted lines indicate error. Scale bar: 25 μm (A).

Guam ALS/PDC is characterized by the presence of dense and widely distributed cortical NFTs affecting motor, sensory, and association areas of the cerebral cortex (11). Chronic dietary exposure to BMAA triggered cortical NFTs in the vervet with a density and distribution similar to Guam ALS/PDC (17, 18, 50). To investigate the relationship between BMAA-induced cortical and spinal cord pathologies, the median tau AT8^+^ NFT density in the same vervets was calculated across seven cortical brain regions. Chronic BMAA exposure increased the median tau AT8^+^ NFT density by 3.1-fold (p ≤ 0.0001) and was attenuated 40% by coadministration of l-serine (Fig. 8A, B). Cortical AT8^+^ NFT density deposition observed in BMAA-dosed vervets was also positively correlated with the concentration of BMAA detected in the spinal cord as a marker of exposures (r = 0.6804, p *=* 0.0004) (Fig. 8C) and the ALS/MND-type pathology observed in descending white matter tracts and the anterior horns. Cortical AT8^+^ NFT density was positively correlated with the total area of IbA1^+^ microglia in the lateral cortical spinal tract (r = 0.6399, p *=* 0.0004) (Fig. 8D), the density and distribution of TDP-43^+^ cytoplasmic inclusion in motor neurons (r = 0.4905, p *=* 0.01) (Fig. 8E), and the number of reactive GFAP^+^ astroglia adjacent to those motor neurons (r = 0.6488, p *=* 0.0003) (Fig. 8F). In all these correlation analyses, coadministration of l-serine decreased the degree of the ALS/MND-type pathological changes observed in the cerebral cortex, anterior horn, and lateral corticospinal spinal tracts (Fig. 8C–F). These observations suggest that chronic dietary exposure to BMAA induces degeneration of upper and lower motor neurons in vervets and coadministration of l-serine reduces these pathological changes.

## DISCUSSION

We report here that chronic dietary exposure to the cyanotoxin BMAA induces ALS/MND-type degeneration of the upper and lower motor neurons in a vervet model. This includes protein inclusions in anterior horn motor neurons, lower motor neuron degeneration, reactive astrogliosis, and microglial activation characteristic of axonal damage and loss of myelinated fibers in the primary descending motor pathways of the vervet spinal cord. The vervet BMAA toxin exposure model recapitulates the features of Guam ALS/PDC and ALS/MND neuropathology including degeneration of both upper and lower motor neurons and TDP-43^+^ neuronal inclusions (43, 51). It is noteworthy to mention that the vervet carries the APOE4 genotype (52), which may explain the prevalence of cortical AT8^+^ NFTs, in keeping with the cortical dementia associated with Guam ALS/PDC (11, 53). These data suggest that BMAA-exposed vervets may serve as a useful experimental model for testing novel therapeutics for the treatment of ALS/MND.

In the vervet, l-serine attenuates the occurrence of NFTs in upper motor neurons of the primary motor cortex (18). In the spinal cord, we observed rare NFTs in BMAA-fed vervets, consistent with what has been observed in Guamanian ALS/PDC patients (53). In addition, we found evidence of other neuronal protein inclusions characteristic of ALS/MND, including TDP-43^+^, FUS^+^, UBIQ^+^, and Bunina bodies. l-Serine coadministration with BMAA reduced the number of anterior horn neuron protein inclusions, microglial activation, and reactive astrogliosis. Moreover, l-serine protected against the overall development of cortical NFTs and reduced pathology leading to axonal damage in the lateral corticospinal tracts in the vervet BMAA model.

In vitro, l-serine inhibits misincorporation of BMAA into proteins (54) and modulates the endoplasmic reticulum unfolded protein response (55). In our vervet study, coadministration of l-serine did not reduce the amount of free BMAA nor did it decrease the detection of BMAA measured in the protein fraction. This observation suggests that alternative mechanisms of neuroprotection, unrelated to protein misfolding or dysfunction may explain the results observed in vervets . Other possible mechanisms for l-serine neuroprotection include proliferation of oligodendrocytes stimulating myelin repair (56) or restoring serine homeostasis through racemase conversion of l- to d-serine (57). l-Serine uptake by astroglia may lead to a compensatory increase in d-serine following dietary BMAA exposures. d-Serine is a glutamate antagonist at AMPA/kainate receptors, which could offset the excitotoxic effects of BMAA binding to NMDA receptors on motor neurons (27, 58, 59).

l-Serine has been suggested to have therapeutic benefit for alleviating symptoms associated with some neurological diseases (56, 57, 60–63). Currently, a phase IIa trial in ALS/MND patients for tolerability and efficacy of l-serine is underway (ClinicalTrials.gov Identifier: NCT03580616). Thus, pharmacological modulation of the serine pathway presents a promising therapeutic approach for treatment of ALS/MND. Our results provide preclinical evidence of the efficacy of l-serine and suggest that the BMAA exposure to vervets may be a useful model for testing compounds to slow or reverse the progression of motor neuron damage. Additional studies are needed in the vervet to determine the critical window and dose-related effects of l-serine when combined with BMAA exposure as well as the potential effects of augmenting l-serine to the diet after discontinuation of chronic BMAA exposures.

In conclusion, we demonstrate that chronic dietary exposure to the cyanobacterial toxin BMAA causes degeneration of the upper and lower motor neurons, activates microglia in the lateral corticospinal tracts, and induces proteinopathies with reactive astrogliosis in the anterior horns of the spinal cord that are characteristic of ALS/MND. The BMAA-dosed vervet can be used to model the neuropathology of Guam ALS/PDC and ALS/MND. The reduction of BMAA triggered pathology by l-serine supports the use of this amino acid as a therapeutic intervention to slow progression of the early stages of ALS/MND.

## Supplementary Material

nlaa002_Supplementary_Data

## Notes

Josephine P. and John J. Louis Foundation William Stamps Farish Fund Patrick and Heather Henry University of Miami Brain Endowment Bank, the Brain Research Fund, which was funded by a generous donation from Dr. Rita Eisenberg.

The Brain Chemistry Labs has a patent pending on the vervet BMAA model for testing drugs to prevent progressive neurodegeneration (PAC, SAB, JSM, and DCM).
