# Supplementary material for: l-Serine Reduces Spinal Cord Pathology in a Vervet Model of Preclinical ALS/MND
Source: J Neuropathol Exp Neurol. 2020 Jan 21;79(4):393–406. doi: 10.1093/jnen/nlaa002 (PMC7092359; doi:10.1093/jnen/nlaa002)
Supplement: nlaa002_Supplementary_Data [file nlaa002_supplementary_data.zip › nlaa002-Suppl_Data/Davis 2019 JNEN Figure S3 01 24 20[AU].docx]

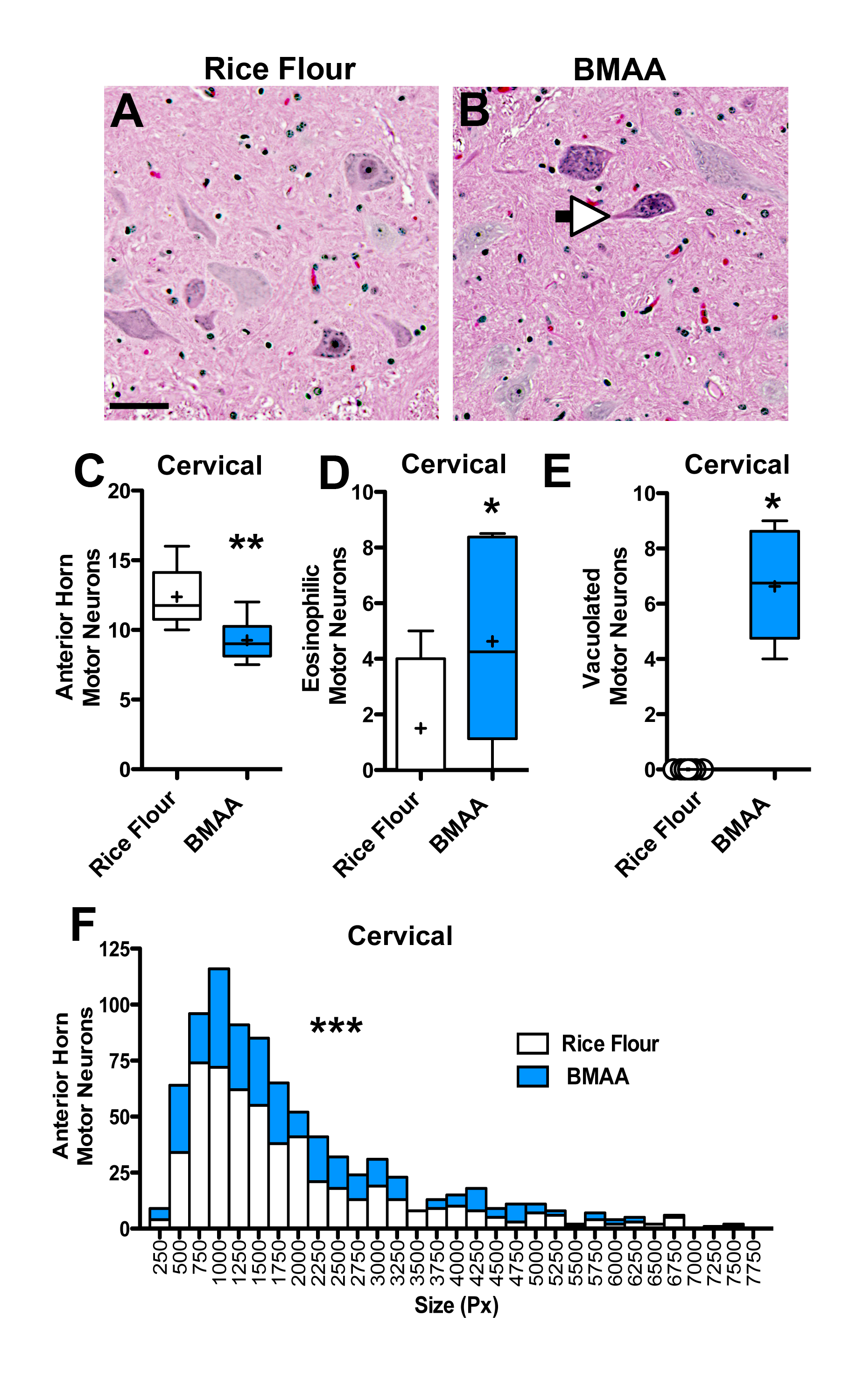


**Figure S3. Anterior Horn Motor Neuron Loss:** (**A**) Anterior horn motor neurons from rice flour controls displayed normal cellular architecture and distribution. (**B**) Vervets dosed with BMAA (210 mg/kg/day) exhibited anterior horn motor neurons that were atrophic, sparse with numerous cytoplasmic vacuoles and thinning of Nissl substances. Quantitative analysis of H&E stained tissue sections from cervical spinal cord segments showed (**C**) anterior horn motor neurons were decreased by 23% (**, *P*=0.0016, n=8) (**D**) with a 4.3-fold increase in number of eosinophilic neurons (*, *P*=0.016; n=8) and (**E**) numerous cytoplasmic vacuoles (*, *P*<0.0001; n=8). (**F**) Histogram showing the number and size distribution of anterior horn neurons in rice flour and BMAA-dosed vervets. BMAA-dosed vervets had a decreased number and size distribution of neurons after 140-days of oral dosing compared to rice flour controls (***, *P*<0.0001; n=8). Scale bar: 100μM (A, B)
