# Supplementary material for: l-Serine Reduces Spinal Cord Pathology in a Vervet Model of Preclinical ALS/MND
Source: J Neuropathol Exp Neurol. 2020 Jan 21;79(4):393–406. doi: 10.1093/jnen/nlaa002 (PMC7092359; doi:10.1093/jnen/nlaa002)
Supplement: nlaa002_Supplementary_Data [file nlaa002_supplementary_data.zip › nlaa002-Suppl_Data/Davis et al JNEN Table S1 1 24 20.docx]

**Supplementary Table 1:** Statistical analysis of data sets

| **Comparison** | **Measure** | **Groups** | **n=** | **Test** | **Figure/ Table** | **Fold Inc.** | ***P*-Value** | **Effect Size (f)** | **r^2^** | **r** |
| --- | --- | --- | --- | --- | --- | --- | --- | --- | --- | --- |
| Multiple | GFAP^+^ No. **^M^** | Cervical (all dosing cohorts) | 8 | ANOVA | 4J | 1.1 | 0.427 ***^NS^*** | 0.272 | 0.078 | --- |
| Multiple | GFAP^+^ No. **^M^** | Lumbar (all dosing cohorts) | 8 | ANOVA | 4K | 1.4 | 0.008 | 0.713 | 0.367 | --- |
| Multiple | GFAP^+^ Total Area | Lumbar (all dosing cohorts) | 8 | Kruskal-Willis | 4L | 0.8^✚^ | 0.008 | 0.779 | --- | --- |
| Multiple | IbA1^+^ Density Ct | Cervical (all dosing cohorts) | 8 | ANOVA | 6D | 1.7 | 0.048 | 0.542 | 0.252 | --- |
| Multiple | IbA1^+^ Total Area | Cervical (all dosing cohorts) | 8 | ANOVA | 6E | 1.6 | 0.011 | 0.686 | 0.349 | --- |
| Multiple | IbA1^+^ Size | Cervical (all dosing cohorts) | 8 | ANOVA | 6F | 1.3 | 0.260 ***^NS^*** | 0.333 | 0.120 | --- |
| Multiple | CD68^+^ Nodule Size | Spinal Cord (all dosing cohorts) | 8 | Kruskal-Willis | 6G | 1.2 | < 0.0001 | 0.244 | --- | --- |
| Multiple | CD68^+^ Int. | Spinal Cord (all dosing cohorts) | 8 | ANOVA | 6H | 0.8^✚^ | 0.010 | 0.691 | 0.353 | --- |
| Multiple | AT8^+^ Density | Cortex (all dosing cohorts) | 8 | ANOVA | 8B | 3.1 | < 0.0001 | 3.071 | 0.899 | --- |
| Correlation | Total [BMAA] vs. AT8^+^ Ct | Spinal Cord vs. Cortex (all dosing cohorts) | 23 | Spearmen | 8C | --- | 0.0004 | --- | --- | 0.680 |
| Correlation | AT8^+^ Ct vs. IbA1^+^ Area | Cortex vs. LCST (all dosing cohorts) | 24 | Spearmen | 8D | --- | 0.0004 | --- | --- | 0.634 |
| Correlation | AT8^+^ Ct vs. TDP43 **^M^** | Cortex vs. Ant. Horn (all dosing cohorts) | 24 | Spearmen | 8E | --- | 0.01 | --- | --- | 0.490 |
| Correlation | AT8^+^ Ct vs. GFAP^+^ No. **^M^** | Cortex vs. Ant. Horn (all dosing cohorts) | 24 | Pearson | 8F | --- | 0.0003 | --- | 0.421 | 0.649 |
| Single | H&E Neurons No. **^M^** | Cervical (Rice Flour vs. BMAA) | 8 | *t*-Test | S3C | 0.8 | 0.0016 | 0.888 | 0.474 | --- |
| Single | H&E Eosinophilic No. **^M^** | Cervical (Rice Flour vs. BMAA) | 8 | *t*-Test | S3D | 4.3**^Δ^** | 0.0240 | 0.634 | 0.251 | --- |
| Single | H&E Vacuoles No. **^M^** | Cervical (Rice Flour vs. BMAA) | 8 | Mann Whitney | S3E | *nac* | 0.0002 | 2.437 | --- | --- |
| Single | H&E Neuron Size | Cervical (Rice Flour vs. BMAA) | 8 | Wilcoxon Mat. | S3F | 0.6 | < 0.0001 | 0.208 |  |  |
| Single | Hydro Pellet [BMAA] | Spinal Cord (BMAA cohorts) | 4/8^⌘^ | Mann Whitney | Tab 1 | 0.9^✚^ | 0.367 ***^NS^*** | 0.318 | --- | --- |
| Single | Free [BMAA] | Spinal Cord (BMAA cohorts) | 5/8^⌘^ | Mann Whitney | Tab 1 | 1.1^✚^ | 0.864 ***^NS^*** | 0.042 | --- | --- |
| Single | Hydro Supernatant [BMAA] | Spinal Cord (BMAA cohorts) | 7/8^⌘^ | Mann Whitney | Tab 1 | 1.7^✚^ | 0.8665 ***^NS^*** | 0.204 | --- | --- |
| Single | TDP43 Inclusions **^M^** | Spinal Cord (Rice Flour vs. BMAA) | 8/8 | Mann Whitney | Tab 2 | 5.0 **^Δ^** | 0.024 | 0.594 | --- | --- |
| Single | TDP43 Inclusions **^M^** | Spinal Cord (BMAA cohorts) | 8/8^⌘^ | Mann Whitney | Tab 2 | 0.8^✚^ | 0.500 ***^NS^*** | 0.073 | --- | --- |
| Multiple | IbA1^+^ Density Ct | Lumbar (all dosing cohorts) | 8 / 8 / 4^⌘^ | Kruskal-Willis | Tab 3 | 0.9 | 0.4169 ***^NS^*** | 0.307 | --- | --- |
| Multiple | IbA1^+^ Size | Lumbar (all dosing cohorts) | 8 / 8 / 4^⌘^ | Kruskal-Willis | Tab 3 | 0.9 | 0.9598 ***^NS^*** | 0.165 | --- | --- |
| Multiple | IbA1^+^ Total Area | Lumbar (all dosing cohorts) | 8 / 8 / 4^⌘^ | Kruskal-Willis | Tab 3 | 1.1 | 0.1346 ***^NS^*** | 0.537 | --- | --- |
| Multiple | IbA1^+^ Total Area | Cervical vs. Lumbar (Rice Flour vs. BMAA) | 8 | RP-ANOVA | Tab 3 | 1.5* | 0.0039 | 0.723 | 0.464 | --- |
| Multiple | LFB Score | Spinal Cord (all dosing cohorts) | 8 | Kruskal-Willis | Text | *nac* | 0.0956 ***^NS^*** | 0.436 | --- | --- |
| Multiple | GFAP^+^ No. **^M^** | Cervical vs. Lumbar (Rice Flour vs. BMAA) | 8 | RP-ANOVA | Text | 1.4* | 0.0009 | 0.792 | 0.538 | --- |
| Single | Luxol Fast Blue Score **^M^** | Spinal Cord (all dosing cohorts) | 8 | Kruskal-Willis | Text | *nac* | 0.0956 ***^NS^*** | 0.436 | --- | --- |
| Correlation | Free vs. Bound [BMAA] | Spinal Cord (BMAA cohorts) | 12 | Spearmen | Text | --- | < 0.0001 | --- | --- | 0.919 |

**Ant.:** anterior; **Ct**: counts (automated); **Inc.**: increase in median; **Int.**: intensity; **LCST:** lateral corticospinal tract; **^M^**: manual scoring; **n**: sample size; ***nac***: unable to calculate; **No.:** number; (**Px**): pixels; **^NS^**: not significant**; RP:** repeated measures; **Δ**: change in median (BMAA vs. rice flour); *: change in median (BMAA lumbar vs. BMAA cervical); **^⌘^**: BMAA + l-Serine cohort; ^✚^: change in median (BMAA + l-Serine vs. BMAA)
