# Supplementary material for: l-Serine Reduces Spinal Cord Pathology in a Vervet Model of Preclinical ALS/MND
Source: J Neuropathol Exp Neurol. 2020 Jan 21;79(4):393–406. doi: 10.1093/jnen/nlaa002 (PMC7092359; doi:10.1093/jnen/nlaa002)
Supplement: nlaa002_Supplementary_Data [file nlaa002_supplementary_data.zip › nlaa002-Suppl_Data/Davis et al 2019 JNEN Figure S1 01 24 20.docx]

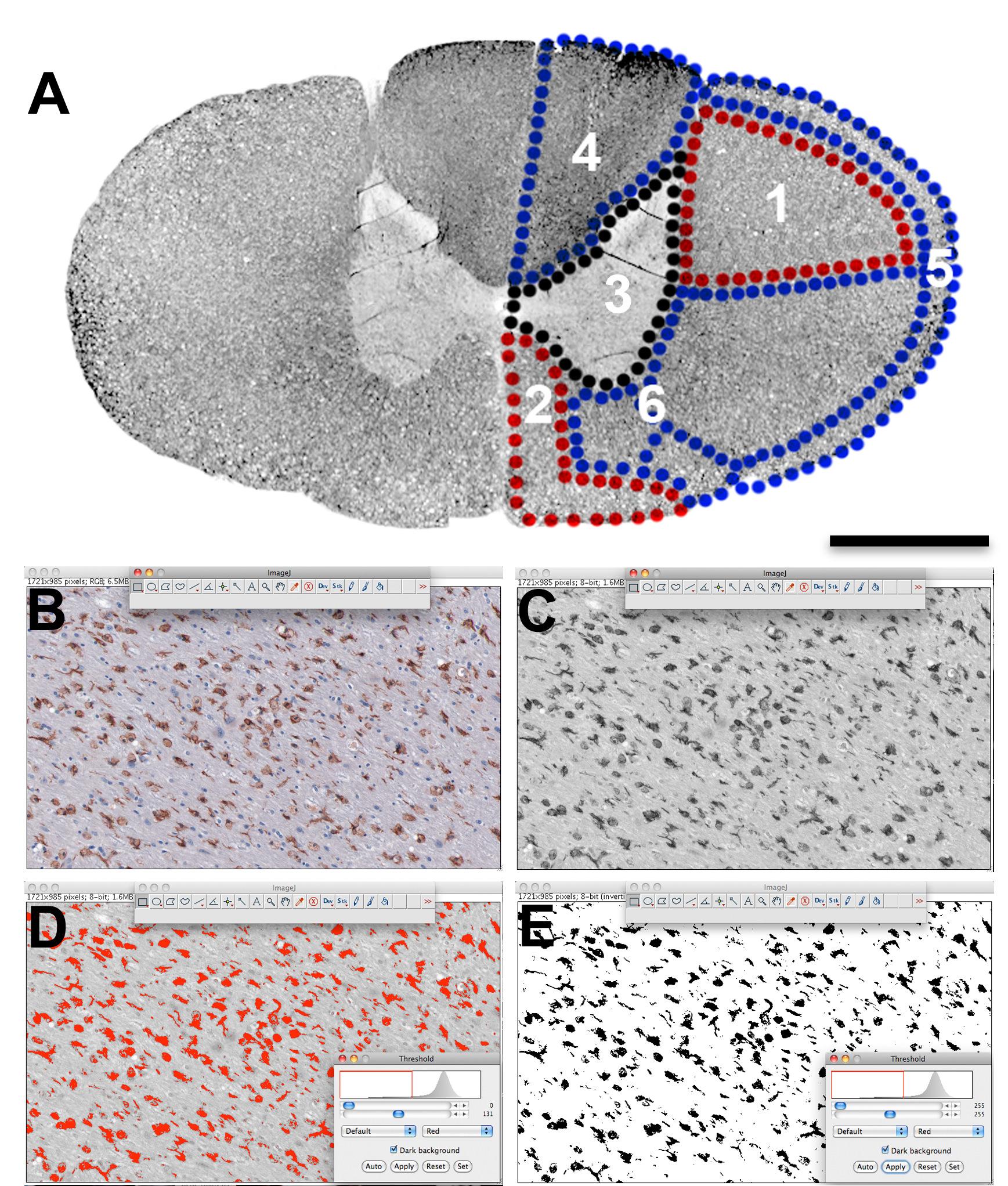


**Figure S1.** **Digital Pathology and Image Analysis:** (**A**) Representative cervical spinal cord segment with unilateral illustration of both efferent (motor, red) and afferent (sensory, blue) pathways. Six anatomical regions of interests were examined in our study: (**1**) lateral corticospinal tracts, (**2**) anterior corticospinal tracts, (**3**) anterior & posterior horns, (**4**) dorsal column medial lemniscus system, (**5**) spinocerebellar tracts and (**6**) anterolateral system. Manual numeration and scoring and subsequent application of an unbiased threshold (ImageJ Ver1.44o software; National Institute of Health) were used to determine changes in cellular morphology and pathology bilaterally in the six regions listed above. For automated analysis, standardize digital images (1721 x 985 pixels or 3259 x 1174 pixels), 6 from 2 spinal cord sections, totaling 12 images per vervet primate, were analyzed. (**B**) Representative image of IbA1^+^ immunostaining of a patient with sporadic ALS/MND. (**C**) Images were converted to 8-bit gray scale allowing for the application of a threshold (**D**), which selects predominately Iba1^+^ microglia cell bodies and processes over background staining. (**E**) After application of the threshold, the image was converted to a binary tiff file allowing for automated quantification of selected microglia per region of interest that are above threshold.
