# Supplementary material for: l-Serine Reduces Spinal Cord Pathology in a Vervet Model of Preclinical ALS/MND
Source: J Neuropathol Exp Neurol. 2020 Jan 21;79(4):393–406. doi: 10.1093/jnen/nlaa002 (PMC7092359; doi:10.1093/jnen/nlaa002)
Supplement: nlaa002_Supplementary_Data [file nlaa002_supplementary_data.zip › nlaa002-Suppl_Data/Davis et al 2019 JNEN Figure S2 1 24 20[AU].docx]

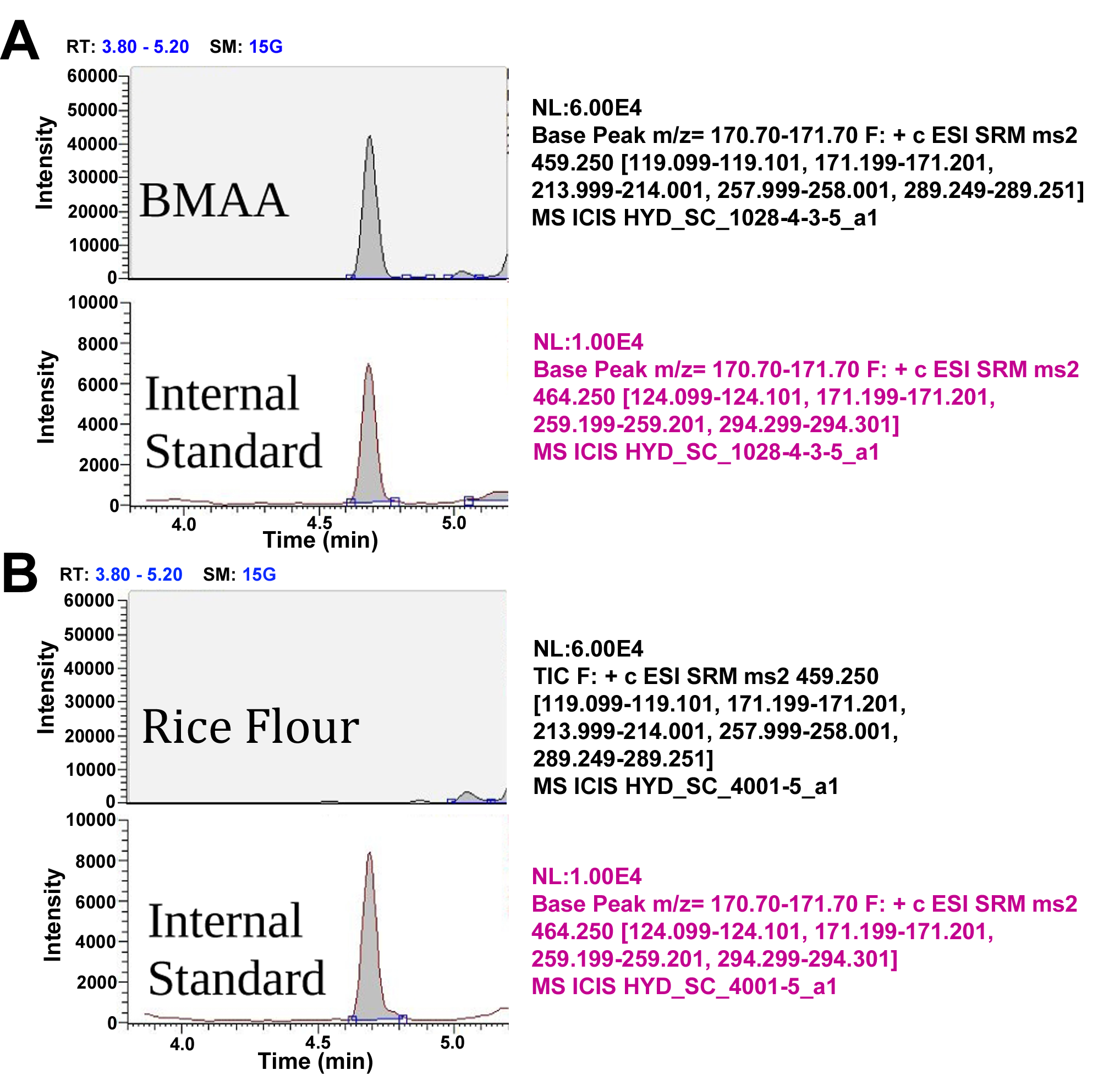


**Figure S2. BMAA Toxin Detection:** (**A**) Tandem mass spectrometry of hydrolyzed spinal cord tissues from a vervet dosed with the cyanobacterial toxin BMAA (210 mg/kg/day) for 140 days. Top panel demonstrates a chromatograph of BMAA (459 m/z) with a retention time of 4.7 min. The bottom panel displays a chromatograph of an internal BMAA standard control. (**B**) Control vervet dosed with rice flour (210 mg/kg/day) for 140 days. The BMAA toxin was not detected. The bottom panels show a BMAA internal standard (464 m/z) chromatograph with retention time of 4.7 min.
