# Supplementary material for: l-Serine Reduces Spinal Cord Pathology in a Vervet Model of Preclinical ALS/MND
Source: J Neuropathol Exp Neurol. 2020 Jan 21;79(4):393–406. doi: 10.1093/jnen/nlaa002 (PMC7092359; doi:10.1093/jnen/nlaa002)
Supplement: nlaa002_Supplementary_Data [file nlaa002_supplementary_data.zip › nlaa002-Suppl_Data/Davis et al 2019 JNEN Figure S4 10 29 19.docx]

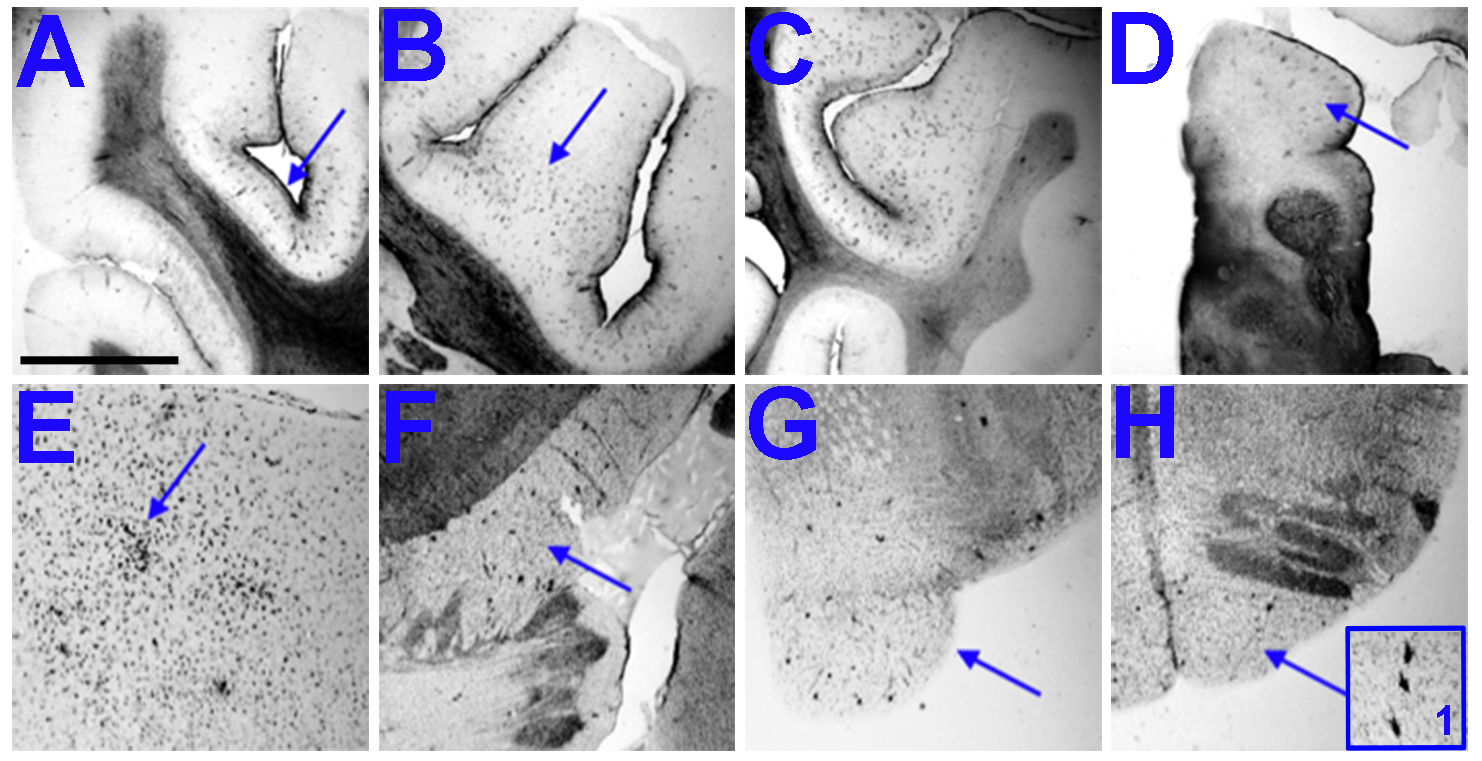


**Figure S4**. **Cortical, Midbrain and Brainstem Glial Activation:** Representative low power light microscopic images of cerebral cortex and midbrain from BMAA-dosed vervets immunoprobed with anti-GFAP a marker for astroglia (**A-D**) and anti-Iba1 a marker for microglia (**E-H**). Reactive astrogliosis is observed in the motor cortex (**A**) frontal cortex **(B**), insular cortex (**C**) and midbrain (**D**). IbA1^+^ microglial nodules can be seen in occipital cortex (**E**), the cerebral peduncles (**F**) and the medullary pyramids **(G-H**). (**Insert 1**) shows clusters of large Iba1^+^ microglial nodules. Scale bar: 4500 μm (A-D), 1000 μm (E-H)
